# Supplementary material for: Identification of photoperiod-induced specific miRNAs in the adrenal glands of Sunite sheep (Ovis aries)
Source: Front Vet Sci. 2022 Jul 22;9:888207. doi: 10.3389/fvets.2022.888207 (PMC9354845; doi:10.3389/fvets.2022.888207)
Supplement: Supplementary file 1 [file Data_Sheet_1.ZIP › Supplementary file/miRDeep2 file.zip!/miRDeep2 file/Novel_Structure/SP42A1/Novel_609.pdf]

Provisional ID	:	NC_019482.2_25262
Score total	:	1.1
Score for star read(s)	:	-1.3
Score for read counts	:	0
Score for mfe	:	0.8
Score for randfold	:	1.6
Score for cons. seed	:	
Total read count	:	28
Mature read count	:	28
Loop read count	:	0
Star read count	:	0

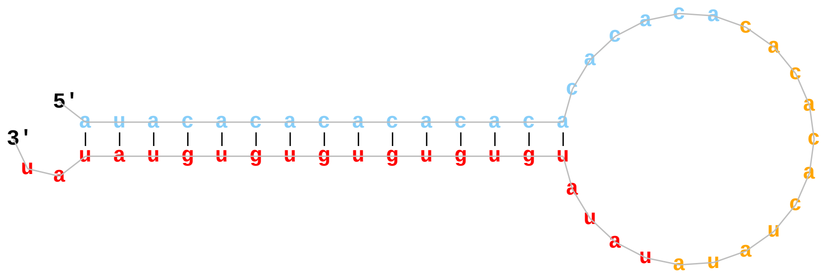

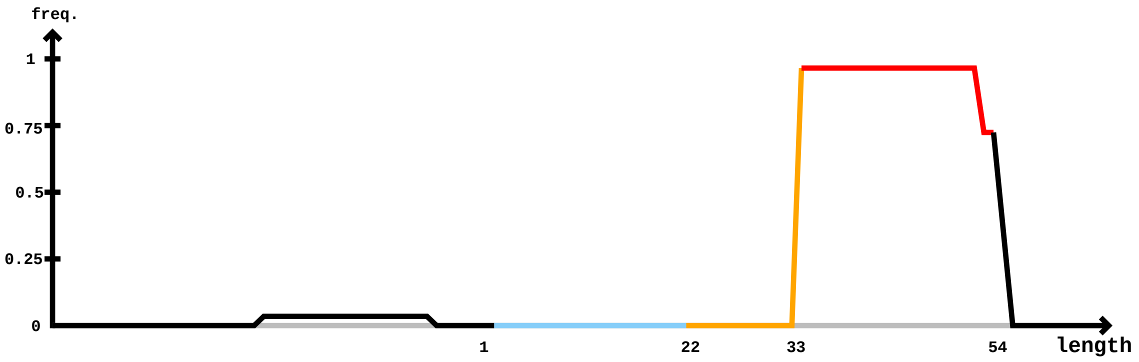

Star Mature

```
5'- cucagggauugaacccaggucuccuguguuuccucauugcaggcauacacacacacacacacacacacacacuauauauauguguguguguguauaucuauauacaca   -3'   exp
    ....((((...(((.((((...)))).)))))))......(((.((((((((((((((....................)))))))))))))).))).........   reads   mm   sample
    ....................cuccuguguuuccucauu......................................................................   1   0   seq
    ............................................................................uauaugugugu Auguguau.............   7   1   seq
    ............................................................................uauaugugugu Auguguauau...........   21   1   seq
```
